# Methyl 3-[(1*H*-benzimidazol-1-yl)meth­yl]-1-methyl-4-(4-methyl­phen­yl)-2′-oxopyrrolidine-2-spiro-3′-1-benzimidazole-3-carboxyl­ate

**DOI:** 10.1107/S1600536810035312

**Published:** 2010-09-04

**Authors:** S. Selvanayagam, B. Sridhar, K. Ravikumar, S. Kathiravan, R. Raghunathan

**Affiliations:** a Department of Physics, Kalasalingam University, Krishnankoil 626 190, India; b Laboratory of X-ray Crystallography, Indian Institute of Chemical Technology, Hyderabad 500 007, India; c Department of Organic Chemistry, University of Madras, Guindy Campus, Chennai 600 025, India

## Abstract

In the title compound, C_29_H_28_N_4_O_3_, the pyrrolidine ring adopts a twist conformation whereas the oxindole and benzimidazole residues are approximately planar with maximum deviations of 0.159 (1) and 0.011 (1) Å, respectively. The oxindole residue is almost perpendicular to the benzimidazole residue, making a dihedral angle of 89.2 (1)°. The methyl-substituted benzene ring is oriented at angles of 47.7 (1) and 71.0 (1)°, respectively, with respect to the oxindole and benzimidazole residues. An intra­molecular C—H⋯O hydrogen bond is observed. In the crystal, mol­ecules associate *via* N—H⋯N hydrogen bonds, forming *R*_2_^2^(9) dimers.

## Related literature

For general background to pyrrolidine derivatives, see: Obniska *et al.* (2010 ▶); Morais *et al.* (2009 ▶); Bello *et al.* (2010 ▶); Moreno-Clavijo *et al.* (2009 ▶); Cheng *et al.* (2008 ▶). For related structures, see: Aravindan *et al.* (2004 ▶); Selvanayagam *et al.* (2005 ▶); Seshadri *et al.* (2003 ▶). For ring-puckering parameters, see: Cremer & Pople (1975 ▶).
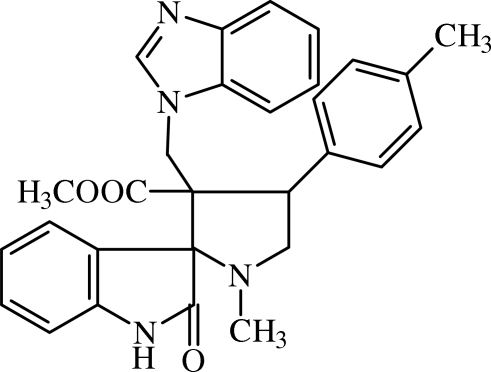


## Experimental

### 

#### Crystal data

C_29_H_28_N_4_O_3_
*M* *_r_* = 480.55
Triclinic, 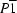

*a* = 9.7605 (5) Å
*b* = 11.2823 (6) Å
*c* = 12.2333 (7) Å
α = 79.960 (1)°
β = 69.539 (1)°
γ = 85.358 (1)°
*V* = 1242.53 (12) Å^3^
*Z* = 2
Mo *K*α radiation
μ = 0.09 mm^−1^
*T* = 292 K
0.26 × 0.24 × 0.22 mm

#### Data collection

Bruker SMART APEX CCD area-detector diffractometer
14287 measured reflections
5705 independent reflections
5041 reflections with *I* > 2σ(*I*)
*R*_int_ = 0.017

#### Refinement

*R*[*F* ^2^ > 2σ(*F* ^2^)] = 0.046
*wR*(*F* ^2^) = 0.131
*S* = 1.04
5705 reflections
328 parameters
H-atom parameters constrained
Δρ_max_ = 0.26 e Å^−3^
Δρ_min_ = −0.26 e Å^−3^

### 

Data collection: *SMART* (Bruker, 2001 ▶); cell refinement: *SAINT* (Bruker, 2001 ▶); data reduction: *SAINT*; program(s) used to solve structure: *SHELXS97* (Sheldrick, 2008 ▶); program(s) used to refine structure: *SHELXL97* (Sheldrick, 2008 ▶); molecular graphics: *ORTEP-3* (Farrugia, 1997 ▶) and *PLATON* (Spek, 2009 ▶); software used to prepare material for publication: *SHELXL97* and *PLATON*.

## Supplementary Material

Crystal structure: contains datablocks I, global. DOI: 10.1107/S1600536810035312/bt5341sup1.cif

Structure factors: contains datablocks I. DOI: 10.1107/S1600536810035312/bt5341Isup2.hkl

Additional supplementary materials:  crystallographic information; 3D view; checkCIF report

## Figures and Tables

**Table 1:** Hydrogen-bond geometry (Å, °)

*D*—H⋯*A*	*D*—H	H⋯*A*	*D*⋯*A*	*D*—H⋯*A*
N2—H2⋯N4^i^	0.86	2.05	2.888 (2)	165
C16—H16⋯O1	0.93	2.33	3.050 (2)	134

Symmetry code: (i) 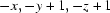.

**Fig. 1.**
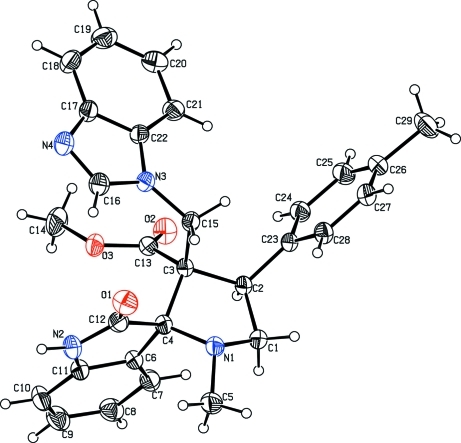
The molecular structure of the title compound, showing the atom-numbering scheme. Displacement ellipsoids are drawn at the 30% probability level

**Fig. 2.**
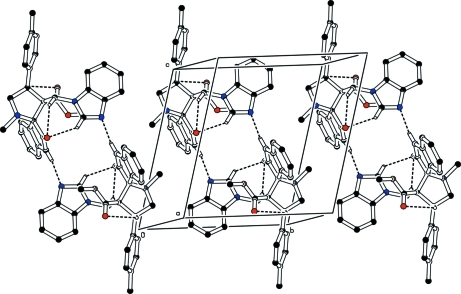
Molecular packing of the title compound, viewed down the a axis; H-bonds are shown as dashed lines. For the sake of clarity, H atoms, not involved in hydrogen bonds, have been omitted

## supplementary crystallographic information

### Comment

Pyrrolidine derivatives possess anticonvulsant (Obniska *et al.*, 2010), anti-angiogenic (Morais *et al.*, 2009) and antitumor (Bello *et al.*, 2010) activities. These derivatives are used as inhibitors of alpha-L-fucosidases (Moreno-Clavijo *et al.*, 2009) and matrix metalloproteinase (Cheng *et al.*, 2008). In view of these importance, we have undertaken the crystal structure determination of the title compound, a pyrrolidine derivative, and the results are presented here.

The molecular structure of (I) is illustrated in Fig. 1. The geometry of the pyrrolidine and oxindole residues of (I) compares well with that reported in other related structures (see, for example, Aravindan *et al.*, 2004; Selvanayagam *et al.*, 2005; Seshadri *et al.*, 2003).

The sum of the angles at N1 of the pyrrolidine ring [334.8°] and N3 of the imidazole ring [359.9°] are in accordance with sp^3^ and sp^2^ hybridizations. Atom O1 is essentially coplanar with the heterocyclic ring to which it is attached, with a deviation of -0.159 (1) Å. Benzimidazole residue is planar with a maximum deviation of -0.011 (1) Å for atom C22. Atom C29 deviates 0.052 (2) Å from the best plane of the methylphenyl ring.

The dihedral angle between the oxindole and benzimidazole residues is 89.2 (1)°. This indicates that the oxindole residue is almost perpendicular to the benzimidazole residue. The methyl phenyl ring is oriented at an angles of 47.7 (1) and 71.0 (1) ° with respect to the oxindole and benzimidazole residues.

The pyrrolidine ring adopts a twist conformation, with puckering parameters (Cremer & Pople, 1975) q_2_ = 0.454 (1) Å and φ = 149.9 (2)°.

The molecular structure is influenced by an intramolecular C—H···O hydrogen bonds. Atom O1 acts as a bifurcated acceptor for two intramolecular C—H···O hydrogen bonds. In the molecular packing, N—H···N hydrogen bonds link inversion-related molecules to form R_2_^2^(9) graph-set dimer (Fig.2 and Table 1).

### Experimental

To a mixture of isatin (1mmol), sarcosine (1mmol) and Baylis-Hillman adduct (1mmol) was added and heated under reflux in methanol (20ml) until the disappearance of the starting materials as evidenced by TLC. The solvent was removed under vacuo. The crude product was subjected to column chromatography using petroleum ether-ethyl acetate as eluent. Single crystals were grown by slow evaporation from methanol.

### Refinement

H atoms were placed in idealized positions and allowed to ride on their parent atoms, with C—H distances of 0.93-0.97 Å and an N—H distance of 0.86 Å, and Uiso(H) = 1.5U_eq_(C) for methyl H and Uiso(H) = 1.2U_eq_(C,N) for all other H atoms.

### Crystal data

**Table:**

C_29_H_28_N_4_O_3_	*Z* = 2
*M**_r_* = 480.55	*F*(000) = 508
Triclinic, *P*1	*D*_x_ = 1.284 Mg m^−^^3^
Hall symbol: -P 1	Mo *K*α radiation, λ = 0.71073 Å
*a* = 9.7605 (5) Å	Cell parameters from 8628 reflections
*b* = 11.2823 (6) Å	θ = 2.0–27.4°
*c* = 12.2333 (7) Å	µ = 0.09 mm^−^^1^
α = 79.960 (1)°	*T* = 292 K
β = 69.539 (1)°	Block, colourless
γ = 85.358 (1)°	0.26 × 0.24 × 0.22 mm
*V* = 1242.53 (12) Å^3^	

### Data collection

**Table:**

Bruker SMART APEX CCD area-detector diffractometer	5041 reflections with *I* > 2σ(*I*)
Radiation source: fine-focus sealed tube	*R*_int_ = 0.017
graphite	θ_max_ = 28.0°, θ_min_ = 1.8°
ω scans	*h* = −12→12
14287 measured reflections	*k* = −14→14
5705 independent reflections	*l* = −15→16

### Refinement

**Table:**

Refinement on *F*^2^	Primary atom site location: structure-invariant direct methods
Least-squares matrix: full	Secondary atom site location: difference Fourier map
*R*[*F*^2^ > 2σ(*F*^2^)] = 0.046	Hydrogen site location: inferred from neighbouring sites
*wR*(*F*^2^) = 0.131	H-atom parameters constrained
*S* = 1.04	*w* = 1/[σ^2^(*F*_o_^2^) + (0.0777*P*)^2^ + 0.1893*P*] where *P* = (*F*_o_^2^ + 2*F*_c_^2^)/3
5705 reflections	(Δ/σ)_max_ < 0.001
328 parameters	Δρ_max_ = 0.26 e Å^−^^3^
0 restraints	Δρ_min_ = −0.26 e Å^−^^3^

### Special details

**Table:**

Geometry. All esds (except the esd in the dihedral angle between two l.s. planes) are estimated using the full covariance matrix. The cell esds are taken into account individually in the estimation of esds in distances, angles and torsion angles; correlations between esds in cell parameters are only used when they are defined by crystal symmetry. An approximate (isotropic) treatment of cell esds is used for estimating esds involving l.s. planes.
Refinement. Refinement of F^2^ against ALL reflections. The weighted R-factor wR and goodness of fit S are based on F^2^, conventional R-factors R are based on F, with F set to zero for negative F^2^. The threshold expression of F^2^ > 2sigma(F^2^) is used only for calculating R-factors(gt) etc. and is not relevant to the choice of reflections for refinement. R-factors based on F^2^ are statistically about twice as large as those based on F, and R- factors based on ALL data will be even larger.

### Fractional atomic coordinates and isotropic or equivalent isotropic displacement parameters (Å^2^)

**Table:**

	*x*	*y*	*z*	*U*_iso_*/*U*_eq_	
O1	−0.02562 (10)	0.75938 (10)	0.40536 (9)	0.0564 (2)	
O2	0.45463 (10)	0.62452 (9)	0.06224 (9)	0.0556 (2)	
O3	0.30370 (10)	0.55640 (7)	0.24567 (8)	0.0475 (2)	
N1	0.19339 (11)	0.93029 (8)	0.24498 (9)	0.0415 (2)	
N2	0.16540 (13)	0.69375 (10)	0.47180 (9)	0.0498 (3)	
H2	0.1161	0.6547	0.5395	0.060*	
N3	0.05799 (10)	0.60212 (9)	0.17842 (8)	0.0392 (2)	
N4	−0.04307 (15)	0.42908 (11)	0.28856 (10)	0.0592 (3)	
C1	0.30089 (16)	0.97153 (11)	0.12846 (10)	0.0467 (3)	
H1A	0.3882	0.9989	0.1363	0.056*	
H1B	0.2603	1.0371	0.0857	0.056*	
C2	0.33690 (12)	0.86094 (10)	0.06345 (9)	0.0371 (2)	
H2A	0.4416	0.8431	0.0465	0.045*	
C3	0.25260 (11)	0.75294 (9)	0.15982 (9)	0.0337 (2)	
C4	0.23182 (12)	0.80336 (10)	0.27776 (9)	0.0363 (2)	
C5	0.18078 (18)	1.00891 (12)	0.33182 (12)	0.0546 (3)	
H5A	0.2735	1.0115	0.3421	0.082*	
H5B	0.1087	0.9780	0.4059	0.082*	
H5C	0.1518	1.0886	0.3043	0.082*	
C6	0.36486 (13)	0.77598 (10)	0.31879 (10)	0.0383 (2)	
C7	0.51257 (14)	0.80220 (12)	0.26535 (11)	0.0468 (3)	
H7	0.5466	0.8478	0.1905	0.056*	
C8	0.60970 (16)	0.75910 (14)	0.32568 (14)	0.0560 (3)	
H8	0.7085	0.7767	0.2906	0.067*	
C9	0.55981 (18)	0.69048 (15)	0.43698 (14)	0.0625 (4)	
H9	0.6259	0.6620	0.4753	0.075*	
C10	0.41285 (18)	0.66350 (14)	0.49225 (13)	0.0592 (4)	
H10	0.3794	0.6173	0.5669	0.071*	
C11	0.31760 (14)	0.70791 (11)	0.43221 (10)	0.0438 (3)	
C12	0.10539 (13)	0.74875 (11)	0.39100 (10)	0.0427 (3)	
C13	0.35074 (12)	0.63904 (10)	0.14798 (10)	0.0380 (2)	
C14	0.3828 (2)	0.44195 (14)	0.24091 (17)	0.0713 (5)	
H14A	0.3969	0.4160	0.1670	0.107*	
H14B	0.3277	0.3831	0.3046	0.107*	
H14C	0.4762	0.4509	0.2480	0.107*	
C15	0.10345 (12)	0.72765 (10)	0.14655 (10)	0.0384 (2)	
H15A	0.0272	0.7758	0.1952	0.046*	
H15B	0.1105	0.7551	0.0651	0.046*	
C16	−0.02908 (15)	0.54492 (13)	0.28527 (11)	0.0517 (3)	
H16	−0.0745	0.5843	0.3499	0.062*	
C17	0.04075 (15)	0.40654 (11)	0.17515 (12)	0.0481 (3)	
C18	0.06481 (18)	0.29959 (12)	0.12571 (15)	0.0613 (4)	
H18	0.0230	0.2278	0.1704	0.074*	
C19	0.15226 (18)	0.30483 (13)	0.00910 (16)	0.0616 (4)	
H19	0.1696	0.2351	−0.0251	0.074*	
C20	0.21573 (15)	0.41222 (14)	−0.05946 (14)	0.0554 (3)	
H20	0.2742	0.4120	−0.1379	0.066*	
C21	0.19363 (13)	0.51890 (12)	−0.01339 (11)	0.0461 (3)	
H21	0.2356	0.5903	−0.0590	0.055*	
C22	0.10513 (12)	0.51376 (10)	0.10505 (10)	0.0390 (2)	
C23	0.31253 (12)	0.88215 (10)	−0.05502 (9)	0.0361 (2)	
C24	0.41258 (14)	0.83543 (11)	−0.15145 (11)	0.0440 (3)	
H24	0.4914	0.7881	−0.1418	0.053*	
C25	0.39601 (17)	0.85871 (13)	−0.26221 (11)	0.0536 (3)	
H25	0.4643	0.8269	−0.3252	0.064*	
C26	0.27911 (17)	0.92865 (12)	−0.27996 (11)	0.0505 (3)	
C27	0.17746 (15)	0.97312 (11)	−0.18349 (11)	0.0475 (3)	
H27	0.0969	1.0180	−0.1927	0.057*	
C28	0.19461 (13)	0.95146 (11)	−0.07328 (10)	0.0428 (3)	
H28	0.1263	0.9837	−0.0106	0.051*	
C29	0.2617 (3)	0.95730 (18)	−0.40035 (15)	0.0789 (5)	
H29A	0.3561	0.9562	−0.4609	0.118*	
H29B	0.2167	1.0357	−0.4086	0.118*	
H29C	0.2013	0.8982	−0.4077	0.118*	

### Atomic displacement parameters (Å^2^)

**Table:**

	*U*^11^	*U*^22^	*U*^33^	*U*^12^	*U*^13^	*U*^23^
O1	0.0441 (5)	0.0703 (6)	0.0480 (5)	−0.0038 (4)	−0.0057 (4)	−0.0113 (4)
O2	0.0484 (5)	0.0570 (5)	0.0528 (5)	0.0094 (4)	−0.0084 (4)	−0.0094 (4)
O3	0.0602 (5)	0.0349 (4)	0.0466 (5)	0.0031 (4)	−0.0212 (4)	0.0000 (3)
N1	0.0536 (6)	0.0351 (5)	0.0368 (5)	0.0028 (4)	−0.0177 (4)	−0.0051 (4)
N2	0.0576 (6)	0.0524 (6)	0.0318 (5)	−0.0095 (5)	−0.0096 (4)	0.0048 (4)
N3	0.0406 (5)	0.0383 (5)	0.0365 (5)	−0.0090 (4)	−0.0115 (4)	0.0004 (4)
N4	0.0737 (8)	0.0534 (6)	0.0456 (6)	−0.0246 (6)	−0.0172 (6)	0.0090 (5)
C1	0.0694 (8)	0.0361 (6)	0.0368 (6)	−0.0109 (5)	−0.0216 (5)	0.0000 (5)
C2	0.0427 (5)	0.0368 (5)	0.0314 (5)	−0.0079 (4)	−0.0135 (4)	0.0008 (4)
C3	0.0368 (5)	0.0332 (5)	0.0298 (5)	−0.0030 (4)	−0.0112 (4)	−0.0009 (4)
C4	0.0406 (5)	0.0359 (5)	0.0307 (5)	−0.0023 (4)	−0.0114 (4)	−0.0018 (4)
C5	0.0766 (9)	0.0460 (7)	0.0448 (7)	0.0063 (6)	−0.0237 (6)	−0.0134 (5)
C6	0.0470 (6)	0.0363 (5)	0.0331 (5)	−0.0020 (4)	−0.0163 (4)	−0.0036 (4)
C7	0.0483 (6)	0.0503 (7)	0.0425 (6)	−0.0057 (5)	−0.0179 (5)	−0.0016 (5)
C8	0.0509 (7)	0.0625 (8)	0.0613 (8)	0.0006 (6)	−0.0275 (6)	−0.0106 (7)
C9	0.0697 (9)	0.0665 (9)	0.0634 (9)	0.0109 (7)	−0.0415 (8)	−0.0080 (7)
C10	0.0788 (10)	0.0575 (8)	0.0446 (7)	0.0028 (7)	−0.0319 (7)	0.0039 (6)
C11	0.0566 (7)	0.0398 (6)	0.0351 (6)	−0.0025 (5)	−0.0175 (5)	−0.0017 (4)
C12	0.0463 (6)	0.0436 (6)	0.0343 (5)	−0.0047 (5)	−0.0075 (5)	−0.0070 (4)
C13	0.0406 (5)	0.0375 (5)	0.0389 (5)	−0.0009 (4)	−0.0177 (4)	−0.0046 (4)
C14	0.0865 (11)	0.0433 (7)	0.0834 (11)	0.0160 (7)	−0.0373 (9)	0.0012 (7)
C15	0.0398 (5)	0.0351 (5)	0.0400 (6)	−0.0048 (4)	−0.0158 (4)	0.0010 (4)
C16	0.0561 (7)	0.0544 (7)	0.0385 (6)	−0.0177 (6)	−0.0094 (5)	0.0022 (5)
C17	0.0555 (7)	0.0417 (6)	0.0492 (7)	−0.0115 (5)	−0.0243 (6)	0.0056 (5)
C18	0.0771 (10)	0.0371 (6)	0.0793 (10)	−0.0093 (6)	−0.0426 (8)	0.0029 (6)
C19	0.0688 (9)	0.0489 (7)	0.0816 (11)	0.0077 (6)	−0.0408 (8)	−0.0205 (7)
C20	0.0486 (7)	0.0646 (8)	0.0584 (8)	0.0025 (6)	−0.0205 (6)	−0.0209 (7)
C21	0.0416 (6)	0.0511 (7)	0.0463 (6)	−0.0083 (5)	−0.0150 (5)	−0.0063 (5)
C22	0.0391 (5)	0.0378 (5)	0.0428 (6)	−0.0057 (4)	−0.0184 (5)	−0.0020 (4)
C23	0.0426 (5)	0.0341 (5)	0.0305 (5)	−0.0078 (4)	−0.0121 (4)	0.0003 (4)
C24	0.0506 (6)	0.0419 (6)	0.0395 (6)	0.0020 (5)	−0.0155 (5)	−0.0075 (5)
C25	0.0706 (9)	0.0530 (7)	0.0369 (6)	0.0040 (6)	−0.0159 (6)	−0.0139 (5)
C26	0.0725 (8)	0.0458 (6)	0.0394 (6)	−0.0056 (6)	−0.0270 (6)	−0.0042 (5)
C27	0.0556 (7)	0.0434 (6)	0.0462 (6)	−0.0016 (5)	−0.0243 (6)	0.0003 (5)
C28	0.0449 (6)	0.0432 (6)	0.0368 (6)	−0.0017 (5)	−0.0115 (5)	−0.0019 (5)
C29	0.1203 (16)	0.0809 (11)	0.0495 (8)	0.0099 (11)	−0.0479 (10)	−0.0122 (8)

### Geometric parameters (Å, °)

**Table:**

O1—C12	1.2271 (16)	C9—C10	1.389 (2)
O2—C13	1.2019 (14)	C9—H9	0.9300
O3—C13	1.3437 (14)	C10—C11	1.3888 (18)
O3—C14	1.4492 (16)	C10—H10	0.9300
N1—C5	1.4662 (16)	C14—H14A	0.9600
N1—C1	1.4668 (16)	C14—H14B	0.9600
N1—C4	1.4732 (14)	C14—H14C	0.9600
N2—C12	1.3555 (16)	C15—H15A	0.9700
N2—C11	1.4055 (17)	C15—H15B	0.9700
N2—H2	0.8600	C16—H16	0.9300
N3—C16	1.3657 (15)	C17—C22	1.4065 (16)
N3—C22	1.4000 (16)	C17—C18	1.409 (2)
N3—C15	1.4639 (14)	C18—C19	1.376 (2)
N4—C16	1.3172 (19)	C18—H18	0.9300
N4—C17	1.3981 (19)	C19—C20	1.400 (2)
C1—C2	1.5467 (16)	C19—H19	0.9300
C1—H1A	0.9700	C20—C21	1.3866 (19)
C1—H1B	0.9700	C20—H20	0.9300
C2—C23	1.5247 (14)	C21—C22	1.3973 (17)
C2—C3	1.5921 (14)	C21—H21	0.9300
C2—H2A	0.9800	C23—C24	1.3968 (16)
C3—C13	1.5357 (15)	C23—C28	1.3998 (16)
C3—C15	1.5735 (15)	C24—C25	1.3976 (18)
C3—C4	1.5840 (15)	C24—H24	0.9300
C4—C6	1.5396 (16)	C25—C26	1.393 (2)
C4—C12	1.5658 (15)	C25—H25	0.9300
C5—H5A	0.9600	C26—C27	1.391 (2)
C5—H5B	0.9600	C26—C29	1.5173 (18)
C5—H5C	0.9600	C27—C28	1.3934 (17)
C6—C7	1.3915 (17)	C27—H27	0.9300
C6—C11	1.4021 (16)	C28—H28	0.9300
C7—C8	1.4033 (18)	C29—H29A	0.9600
C7—H7	0.9300	C29—H29B	0.9600
C8—C9	1.386 (2)	C29—H29C	0.9600
C8—H8	0.9300		
			
C13—O3—C14	115.61 (11)	N2—C12—C4	108.20 (10)
C5—N1—C1	113.09 (10)	O2—C13—O3	124.23 (10)
C5—N1—C4	115.51 (9)	O2—C13—C3	124.75 (10)
C1—N1—C4	106.21 (9)	O3—C13—C3	111.01 (9)
C12—N2—C11	112.18 (10)	O3—C14—H14A	109.5
C12—N2—H2	123.9	O3—C14—H14B	109.5
C11—N2—H2	123.9	H14A—C14—H14B	109.5
C16—N3—C22	105.96 (10)	O3—C14—H14C	109.5
C16—N3—C15	128.19 (11)	H14A—C14—H14C	109.5
C22—N3—C15	125.72 (9)	H14B—C14—H14C	109.5
C16—N4—C17	104.88 (11)	N3—C15—C3	116.04 (9)
N1—C1—C2	105.55 (9)	N3—C15—H15A	108.3
N1—C1—H1A	110.6	C3—C15—H15A	108.3
C2—C1—H1A	110.6	N3—C15—H15B	108.3
N1—C1—H1B	110.6	C3—C15—H15B	108.3
C2—C1—H1B	110.6	H15A—C15—H15B	107.4
H1A—C1—H1B	108.8	N4—C16—N3	114.03 (12)
C23—C2—C1	114.32 (9)	N4—C16—H16	123.0
C23—C2—C3	117.51 (9)	N3—C16—H16	123.0
C1—C2—C3	104.77 (8)	N4—C17—C22	109.58 (11)
C23—C2—H2A	106.5	N4—C17—C18	130.71 (12)
C1—C2—H2A	106.5	C22—C17—C18	119.71 (13)
C3—C2—H2A	106.5	C19—C18—C17	117.84 (13)
C13—C3—C15	109.64 (9)	C19—C18—H18	121.1
C13—C3—C4	112.06 (8)	C17—C18—H18	121.1
C15—C3—C4	113.01 (9)	C18—C19—C20	121.77 (14)
C13—C3—C2	109.12 (9)	C18—C19—H19	119.1
C15—C3—C2	112.02 (8)	C20—C19—H19	119.1
C4—C3—C2	100.71 (8)	C21—C20—C19	121.69 (14)
N1—C4—C6	118.20 (9)	C21—C20—H20	119.2
N1—C4—C12	108.16 (9)	C19—C20—H20	119.2
C6—C4—C12	101.17 (9)	C20—C21—C22	116.65 (12)
N1—C4—C3	100.65 (8)	C20—C21—H21	121.7
C6—C4—C3	112.70 (9)	C22—C21—H21	121.7
C12—C4—C3	116.68 (9)	C21—C22—N3	132.09 (11)
N1—C5—H5A	109.5	C21—C22—C17	122.34 (12)
N1—C5—H5B	109.5	N3—C22—C17	105.55 (10)
H5A—C5—H5B	109.5	C24—C23—C28	117.53 (10)
N1—C5—H5C	109.5	C24—C23—C2	120.08 (10)
H5A—C5—H5C	109.5	C28—C23—C2	122.35 (10)
H5B—C5—H5C	109.5	C23—C24—C25	120.96 (12)
C7—C6—C11	118.67 (11)	C23—C24—H24	119.5
C7—C6—C4	133.14 (10)	C25—C24—H24	119.5
C11—C6—C4	108.15 (10)	C26—C25—C24	121.19 (12)
C6—C7—C8	119.29 (12)	C26—C25—H25	119.4
C6—C7—H7	120.4	C24—C25—H25	119.4
C8—C7—H7	120.4	C27—C26—C25	118.01 (11)
C9—C8—C7	120.61 (13)	C27—C26—C29	120.20 (14)
C9—C8—H8	119.7	C25—C26—C29	121.79 (14)
C7—C8—H8	119.7	C26—C27—C28	120.98 (12)
C8—C9—C10	121.15 (13)	C26—C27—H27	119.5
C8—C9—H9	119.4	C28—C27—H27	119.5
C10—C9—H9	119.4	C27—C28—C23	121.32 (11)
C9—C10—C11	117.67 (13)	C27—C28—H28	119.3
C9—C10—H10	121.2	C23—C28—H28	119.3
C11—C10—H10	121.2	C26—C29—H29A	109.5
C10—C11—C6	122.60 (13)	C26—C29—H29B	109.5
C10—C11—N2	127.34 (12)	H29A—C29—H29B	109.5
C6—C11—N2	110.06 (10)	C26—C29—H29C	109.5
O1—C12—N2	126.27 (11)	H29A—C29—H29C	109.5
O1—C12—C4	125.45 (11)	H29B—C29—H29C	109.5
			
C5—N1—C1—C2	161.69 (10)	C6—C4—C12—N2	−4.58 (12)
C4—N1—C1—C2	33.99 (12)	C3—C4—C12—N2	118.07 (11)
N1—C1—C2—C23	124.14 (10)	C14—O3—C13—O2	−2.91 (18)
N1—C1—C2—C3	−5.90 (12)	C14—O3—C13—C3	176.04 (12)
C23—C2—C3—C13	92.40 (11)	C15—C3—C13—O2	104.57 (13)
C1—C2—C3—C13	−139.46 (9)	C4—C3—C13—O2	−129.11 (12)
C23—C2—C3—C15	−29.19 (13)	C2—C3—C13—O2	−18.46 (15)
C1—C2—C3—C15	98.95 (10)	C15—C3—C13—O3	−74.38 (11)
C23—C2—C3—C4	−149.56 (10)	C4—C3—C13—O3	51.94 (12)
C1—C2—C3—C4	−21.42 (11)	C2—C3—C13—O3	162.60 (9)
C5—N1—C4—C6	−50.74 (14)	C16—N3—C15—C3	92.19 (15)
C1—N1—C4—C6	75.51 (12)	C22—N3—C15—C3	−83.07 (13)
C5—N1—C4—C12	63.29 (13)	C13—C3—C15—N3	26.13 (13)
C1—N1—C4—C12	−170.47 (9)	C4—C3—C15—N3	−99.65 (11)
C5—N1—C4—C3	−173.86 (10)	C2—C3—C15—N3	147.43 (9)
C1—N1—C4—C3	−47.62 (11)	C17—N4—C16—N3	−0.14 (17)
C13—C3—C4—N1	156.94 (9)	C22—N3—C16—N4	0.01 (16)
C15—C3—C4—N1	−78.59 (10)	C15—N3—C16—N4	−175.99 (12)
C2—C3—C4—N1	41.06 (10)	C16—N4—C17—C22	0.22 (15)
C13—C3—C4—C6	30.07 (12)	C16—N4—C17—C18	−178.59 (15)
C15—C3—C4—C6	154.54 (9)	N4—C17—C18—C19	178.97 (14)
C2—C3—C4—C6	−85.80 (10)	C22—C17—C18—C19	0.3 (2)
C13—C3—C4—C12	−86.36 (12)	C17—C18—C19—C20	−0.2 (2)
C15—C3—C4—C12	38.11 (13)	C18—C19—C20—C21	0.0 (2)
C2—C3—C4—C12	157.77 (9)	C19—C20—C21—C22	0.17 (19)
N1—C4—C6—C7	−59.69 (17)	C20—C21—C22—N3	−178.63 (12)
C12—C4—C6—C7	−177.48 (13)	C20—C21—C22—C17	−0.07 (18)
C3—C4—C6—C7	57.16 (16)	C16—N3—C22—C21	178.86 (13)
N1—C4—C6—C11	122.37 (11)	C15—N3—C22—C21	−5.02 (19)
C12—C4—C6—C11	4.58 (11)	C16—N3—C22—C17	0.13 (13)
C3—C4—C6—C11	−120.78 (10)	C15—N3—C22—C17	176.25 (10)
C11—C6—C7—C8	0.58 (18)	N4—C17—C22—C21	−179.10 (11)
C4—C6—C7—C8	−177.19 (12)	C18—C17—C22—C21	−0.14 (19)
C6—C7—C8—C9	0.4 (2)	N4—C17—C22—N3	−0.21 (14)
C7—C8—C9—C10	−0.6 (2)	C18—C17—C22—N3	178.75 (11)
C8—C9—C10—C11	−0.1 (2)	C1—C2—C23—C24	139.36 (11)
C9—C10—C11—C6	1.2 (2)	C3—C2—C23—C24	−97.22 (12)
C9—C10—C11—N2	−178.74 (13)	C1—C2—C23—C28	−38.27 (14)
C7—C6—C11—C10	−1.40 (19)	C3—C2—C23—C28	85.16 (13)
C4—C6—C11—C10	176.88 (12)	C28—C23—C24—C25	0.84 (18)
C7—C6—C11—N2	178.53 (11)	C2—C23—C24—C25	−176.90 (11)
C4—C6—C11—N2	−3.18 (13)	C23—C24—C25—C26	−0.3 (2)
C12—N2—C11—C10	179.99 (13)	C24—C25—C26—C27	−1.1 (2)
C12—N2—C11—C6	0.05 (15)	C24—C25—C26—C29	178.44 (15)
C11—N2—C12—O1	−173.75 (12)	C25—C26—C27—C28	2.0 (2)
C11—N2—C12—C4	3.05 (14)	C29—C26—C27—C28	−177.59 (14)
N1—C4—C12—O1	47.38 (15)	C26—C27—C28—C23	−1.45 (19)
C6—C4—C12—O1	172.25 (12)	C24—C23—C28—C27	0.02 (17)
C3—C4—C12—O1	−65.10 (16)	C2—C23—C28—C27	177.70 (10)
N1—C4—C12—N2	−129.44 (10)		

### Hydrogen-bond geometry (Å, °)

**Table:**

*D*—H···*A*	*D*—H	H···*A*	*D*···*A*	*D*—H···*A*
N2—H2···N4^i^	0.86	2.05	2.888 (2)	165
C16—H16···O1	0.93	2.33	3.050 (2)	134

Symmetry codes: (i) −*x*, −*y*+1, −*z*+1.
